# When misunderstanding meets artificial intelligence: the critical role of trust in human–AI and human–human team communication and performance

**DOI:** 10.3389/fpsyg.2025.1637339

**Published:** 2025-10-23

**Authors:** Na Chen, Xinyue Zhang

**Affiliations:** School of Economics and Management, Beijing University of Chemical Technology, Beijing, China

**Keywords:** human–AI teams, artificial intelligence collaboration, misunderstanding types, team trust, communication efficiency, team performance

## Abstract

**Introduction:**

As artificial intelligence (AI) technologies become increasingly integrated into organizational teamwork, managing communication breakdowns in human–AI collaboration has emerged as a significant managerial challenge. Although AI-empowered teams often achieve enhanced efficiency, misunderstandings—especially those caused by AI agents during information exchange—can undermine team trust and impair performance. The mechanisms underlying these effects remain insufficiently explored.

**Methods:**

Grounded in evolutionary psychology and trust theory, this study employed a 2 (team type: human–AI vs. human–human) × 2 (misunderstanding type: information omission vs. ambiguous expression) experimental design. A total of 126 valid participants were assigned to collaboratively complete a planning and writing task for a popular science social media column with their respective teammates.

**Results:**

The findings indicate that information omissions caused by AI agents significantly reduce team trust, which in turn hinders communication efficiency and overall performance. Conversely, the negative impact of ambiguous expressions is moderated by the level of team trust; teams with higher trust demonstrate greater adaptability and resilience. Moderated mediation analyses further reveal that team type influences the dynamic pathway from misunderstanding to trust and performance.

**Discussion:**

This research advances theoretical understanding of misunderstanding management in human–AI teams and provides practical insights for optimizing AI systems and fostering effective human–machine collaboration.

## 1 Introduction

Is artificial intelligence (AI) truly reshaping the way we collaborate in teams? As AI systems increasingly join human teams as “intelligent members,” what new opportunities and challenges arise in human–AI collaboration? In the midst of a sweeping wave of digital transformation, AI not only alters how we live and work but also fundamentally restructures team composition and collaboration mechanisms within organizations. Across sectors such as health care, finance, manufacturing, and education, AI has been widely integrated into teams to support data analysis, decision-making, and innovation. An increasing number of organizations have recognized that AI can enhance operational efficiency and unlock team creativity, thereby generating unprecedented competitive advantages (Jia et al., 2024).

Despite these transformative benefits, the integration of AI into teams foregrounds fundamental questions about the cognitive and social mechanisms underlying effective collaboration—most notably, the evolution and calibration of trust. Evolutionary psychology posits that trust is an adaptive mechanism, shaped by natural selection to facilitate cooperation and mitigate risk within social groups (Cosmides and Tooby, 1992; Barclay, 2004; Glowacki and Lew-Levy, 2022). In ancestral environments, individuals relied on rich social cues—such as facial expressions, vocal tone, and body language—to calibrate trust and coordinate joint action (Tomasello, 2001; Syme and Balliet, 2025). The deployment of AI agents fundamentally disrupts these evolved processes, as AI lacks many of the subtle signals humans have adapted to interpret. This evolutionary mismatch between cognitive adaptations and technologically mediated environments helps explain why trust issues and communication barriers are especially pronounced in human–AI teams (Schelble et al., 2022; Schmutz et al., 2024).

Beyond individual cognition, evolutionary psychology also recognizes the importance of cultural evolution and social learning in shaping collaborative behavior (Boyd and Richerson, 2005; Heyes, 2020; Lew-Levy et al., 2023). Through mechanisms of cultural transmission, humans acquire norms, practices, and interpretive frameworks that facilitate group coordination and meaning-making. In organizational settings, these culturally evolved strategies underpin team dynamics and shared understanding. However, when AI agents join human teams, the lack of shared cultural background and interpretive context can exacerbate cognitive and communicative asymmetries. Compared with traditional human–human teams, human–AI teams differ significantly in terms of member heterogeneity, communication patterns, and coordination dynamics. Compared with human teammates, AI agents process information on the basis of algorithmic logic and big data analytics, which leads to substantial differences in how they think and communicate (Dennis et al., 2023). These cognitive and communicative asymmetries often result in frequent misunderstandings—for example, AI may omit contextually relevant information or deliver suggestions in a manner that lacks human nuance—thereby creating communication breakdowns and cognitive misalignment (Nishant et al., 2024). The opaque nature of AI systems further compounds this issue, as their “black-box” decision-making processes can undermine users’ understanding and trust (Shin, 2021). Consequently, communication obstacles and trust deficits have emerged as critical bottlenecks limiting the performance and effectiveness of human–AI collaboration.

In practice, AI is being deployed in an expanding array of high-value collaborative tasks. In health care, AI assists diagnostic teams to improve decision accuracy and efficiency (Reverberi et al., 2022); in business, strategic teams use AI to analyze complex market data and optimize planning (Carter and Wynne, 2024); and in scientific research, AI accelerates knowledge discovery through large-scale data mining. These use cases demonstrate that human–AI teams can harness complementary strengths to achieve greater collective outcomes. Yet, as AI becomes increasingly embedded in these decision-making processes, misunderstandings between human and AI team members have become more prominent. For instance, AI systems may misinterpret ambiguous human input, fail to grasp nuanced contextual cues, or generate recommendations that conflict with expert intuition (Klingbeil et al., 2024). Such misunderstandings can lead to confusion, inefficiency, or even a breakdown of trust within the team. Nevertheless, persistent challenges related to misunderstandings and trust continue to hinder collaboration, raising a fundamental managerial question: How can AI be transformed from a potential obstacle to a reliable collaborator?

Although interest in AI-augmented teamwork is rapidly growing, the current literature primarily emphasizes task allocation, functional optimization, and performance enhancement. Much less attention has been given to the microlevel interaction mechanisms within human–AI teams. In particular, little is known about how misunderstandings are managed and how trust is built in these hybrid collaborations. Misunderstandings are common in team communication, but they are particularly complex and diverse in human–AI settings. AI agents may fail to deliver complete information due to algorithmic limitations or generate ambiguous expressions that lead to divergent interpretations. These misunderstanding types can disrupt cognitive alignment among team members, erode the foundation of team trust, and ultimately impair communication efficiency and team performance.

Against this backdrop, the present study draws on evolutionary psychology and trust theory to explore how misunderstanding types affect trust and communication in human–AI collaboration. Evolutionary psychology highlights that trust is an adaptive mechanism shaped for cooperation and risk management, while trust theory explains how trust is established, maintained, and can be disrupted in team contexts. In the context of human–AI teams, this perspective highlights how misunderstandings arise not only from technical limitations but also from differences in cognitive processes and the absence of human-like social signals in AI interactions. By situating misunderstandings within this theoretical lens, we can better understand their impact on cognitive coordination and trust-building processes. By examining theoretical gaps and practical dilemmas, we aim to uncover how misunderstandings shape cognitive coordination and trust development in mixed human–AI teams. Specifically, this study contributes to the growing body of research on team collaboration and intelligent systems by providing both conceptual insights and empirical evidence regarding misunderstandings within human–AI teams. It also provides practical guidance for organizations seeking to overcome communication barriers and foster trust in the era of deep human–AI collaboration. Furthermore, this study highlights that the lack of rich social cues in AI-driven interactions can easily lead to trust being miscalibrated—resulting in either excessive reliance on AI agents or unwarranted skepticism. Such miscalibration can undermine communication and team performance, making it crucial to understand and address these challenges in real-world human–AI collaboration. Furthermore, this study emphasizes that the lack of rich social cues in AI-driven interactions can easily result in miscalibrated trust—leading to either excessive reliance on AI agents or unwarranted skepticism. Addressing trust miscalibration is essential for optimizing communication and enhancing team performance in real-world human–AI collaboration.

## 2 Theory and hypotheses

### 2.1 Misunderstanding types and communication efficiency

Communication efficiency is a key determinant of team performance, particularly in collaborative settings (Marlow et al., 2018). Misunderstandings during communication not only disrupt the accurate transmission of information but also undermine interpersonal trust and cooperation (Brewer and Holmes, 2016). Research categorizes misunderstandings in team communication into two primary types: information omission and ambiguous expression (Edwards et al., 2020; Verdonik, 2005). Information omission refers to the failure to convey critical information during communication, resulting in incomplete understanding by the recipient (Doyle and Paton, 2018). In contrast, ambiguous expression arises when the conveyed content is vague or polysemous, leaving room for multiple interpretations (Blott, 2020).

Empirical studies have shown that these two types of misunderstandings have significantly different impacts on communication efficiency (Fiset, 2023). Owing to their more salient cues, information omissions are generally easier to detect and correct through follow-up inquiries or clarification, thus minimizing the duration of communication breakdowns (Brewer and Holmes, 2016; O’Bryan et al., 2024). In contrast, ambiguous expressions are often more covert and may go unnoticed, leading team members to proceed under misaligned assumptions, which results in greater informational distortion and resource waste (Sohrab et al., 2022). Teams facing information omissions are more likely to adopt direct clarification and information-recovery strategies, enhancing overall communication efficiency (Laourou, 2022).

In technologically mediated environments, such as human–AI hybrid teams, the detection and correction of misunderstandings become more complex (Woolley et al., 2023). Importantly, the nature and consequences of misunderstandings differ depending on whether they originate from human or AI participants. Human misunderstandings often stem from subjective interpretation, emotional nuances, or implicit assumptions, which may be quickly recognized and resolved through interactive clarification. In contrast, AI-driven misunderstandings frequently arise from limitations in natural language processing, lack of contextual awareness, or rigid algorithmic logic. These misunderstandings may persist longer or require more explicit intervention, placing greater demands on human team members to detect and address them (Roberts et al., 2022). While AI systems can algorithmically identify some semantic omissions, they still struggle to detect ambiguity effectively in expression (Birhane et al., 2022). Therefore, misunderstandings originating from AI may disrupt communication flow and lower overall team efficiency to a greater extent than those arising from human error. As a result, different misunderstanding types impose varying cognitive and communicative burdens on team members. On this basis, we propose the following hypothesis:

Hypothesis 1a: Communication efficiency is significantly influenced by the type of misunderstanding. Compared with ambiguous expressions, information omissions are more easily identified and corrected, thereby enhancing communication efficiency.

### 2.2 Types of misunderstanding and team performance

Team performance, a central indicator of goal attainment in organizations, is highly dependent on efficient and fluent team communication. Prior research has demonstrated that misunderstanding types exert distinct and significant impacts on team outcomes. While information omissions can cause temporary delays due to incomplete or missing content, they are generally easier to identify and resolve through feedback loops, which limits their long-term detrimental effects on performance (Mahbub et al., 2024). Conversely, ambiguous expressions tend to be more concealed and complex, increasing the risk of systematic deviations in team decisions and actions. These misinterpretations may lead to fundamental disagreements over goals, roles, and strategies, thereby weakening collective output (Klüber et al., 2025). Such misunderstandings can escalate into latent conflicts that erode interpersonal trust and decrease team cohesion and willingness to cooperate, ultimately resulting in project delays, resource waste, or even team dissolution (Malik et al., 2021). In AI-assisted collaboration, structured tasks benefit from the AI’s ability to minimize information omissions (Edelman et al., 2023). However, in unstructured tasks that require nuanced communication and flexible negotiation, the limitations of AI expression make ambiguous misunderstandings particularly problematic (Koçak et al., 2022). Thus, detecting and resolving ambiguous expressions is critical for maintaining performance in human–AI teams. Accordingly, we propose the following hypothesis:

Hypothesis 1b: Team performance is significantly affected by the types of misunderstanding. Compared with ambiguous expressions, information omissions exert a less negative effect on performance.

### 2.3 The mediating role of team trust

Team trust plays a pivotal role in human–AI collaboration, shaping members’ acceptance of AI, willingness to cooperate, and task allocation strategies (McGrath et al., 2025; Dennis et al., 2023). Trust among team members facilitates information sharing and collaborative intent (Imam and Zaheer, 2021) and serves as a buffer against the negative consequences of poor communication (Bahrain et al., 2023). Misunderstandings, however, can undermine trust development within teams. Specifically, information omissions are typically more transparent and easily corrected, thus having a limited impact on trust. In contrast, ambiguous expressions may lead to doubts about the competence or intentions of others—including AI systems—due to their covert nature and interpretive complexity (Schaefer et al., 2017). In human–AI teams, ambiguous misunderstandings further intensify trust challenges between humans and AI, thereby hampering communication and collaboration (Park, 2025). As AI continues to be embedded in team workflows, trust mechanisms become increasingly critical. Studies show that trust in AI directly affects the degree to which its suggestions are accepted and integrated into team decisions (Bedué and Fritzsche, 2022). Moreover, misunderstanding types influence not only interpersonal trust but also trust in AI agents (Li et al., 2024), which subsequently affects communication efficiency and performance. However, empirical models clarifying how different misunderstanding types influence outcomes through trust are still scarce.

While most existing research approaches trust as a social and cognitive construct, recent theoretical developments highlight the importance of its evolutionary origins—especially in novel, technologically mediated environments. From an evolutionary standpoint, trust is not only a cognitive evaluation but also a product of adaptive mechanisms shaped by repeated face-to-face interactions. In ancestral environments, individuals relied on nonverbal cues—such as facial expressions, gestures, and vocal tone—to calibrate trust and facilitate cooperation. The lack of such cues in AI-mediated communication presents a fundamental evolutionary mismatch, potentially undermining the natural calibration of trust. Consequently, misunderstandings in human–AI teams may be more difficult to resolve, as team members cannot rely on the evolved social signals that typically guide trust repair and adjustment.

Therefore, we propose the following hypotheses:

Hypothesis 2a: Team trust mediates the relationship between misunderstanding type and communication efficiency.

Hypothesis 2b: Team trust mediates the relationship between misunderstanding type and team performance.

### 2.4 The moderating role of peer type

The peer type refers to the nature of the interaction partner in a team—human peer versus AI peer—and has been shown to significantly affect team communication and collaboration outcomes (Myers et al., 2010). In human–human teams, shared social norms and past experiences help members efficiently identify and resolve information omissions, thereby mitigating their negative impact on communication (Henningsen and Henningsen, 2007). In human–AI teams, members typically have lower social expectations for AI peers and tend to adapt by taking the initiative to clarify or fill in missing information (Siemon, 2022). However, when dealing with ambiguous expressions, AI’s limitations in understanding unstructured or nuanced input become more apparent (Mahadevkar et al., 2024). In such cases, team members are more likely to attribute communication failure to the cognitive limitations of AI, leading to attributional bias. This bias not only reduces communication efficiency but also undermines trust (Schwartz et al., 2022). In virtual or human–AI teams, the negative effects of ambiguous misunderstandings on trust and performance are particularly pronounced. Moreover, trust in AI is especially vulnerable to ambiguity-induced miscommunication, which subsequently impairs cooperation and performance. Thus, we propose the following hypotheses:

Hypothesis 3a: The relationship between misunderstanding type and communication efficiency is moderated by peer type, with stronger effects on human–AI teams.

Hypothesis 3b: The impact of misunderstanding type on team performance is moderated by peer type; in human–AI teams, ambiguous misunderstandings have stronger negative effects.

Hypothesis 3c: The effect of misunderstanding type on team trust is moderated by peer type; in human–AI teams, ambiguous misunderstandings are more likely to erode trust.

### 2.5 Peer type as a moderator of the trust mediation mechanism

Peer type not only differentiates the interaction dynamics of human–human versus human–AI teams but also influences the mediating role of trust between misunderstanding types and subsequent outcomes. Research has confirmed the central role of trust as a mediator in the link between misunderstanding and both communication efficiency and team performance (Duan et al., 2024; Zhang et al., 2023). However, the strength of this mediation is not uniform across contexts and is significantly moderated by peer type. In human–AI teams, ambiguous misunderstandings are more likely to be attributed to AI limitations, resulting in decreased trust and a magnified negative impact on communication and performance. In contrast, information omissions are often addressed proactively, leading to only minor fluctuations in trust. In human–human teams, regardless of the misunderstanding type, trust can often be restored through social norms and mutual understanding, mitigating the effect on team outcomes (Siemon, 2022). On the basis of these insights, we propose the following hypothesis:

Hypothesis 4: The mediating effect of team trust on the relationships between misunderstanding type and both communication efficiency and team performance is moderated by peer type.

**Figure 1:**
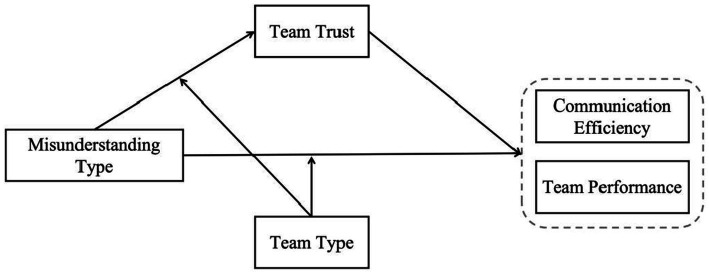
Frame diagram of the research model.

A conceptual model of the research framework is presented in Figure 1.

## 3 Research design

### 3.1 Participants

This study recruited a total of 131 participants with undergraduate or higher educational backgrounds. After excluding participants with academic backgrounds in astronomy and removing invalid data, 126 valid responses were retained for analysis. *A priori* power analysis was conducted using G*Power 3.1 (*α* = 0.05, *f* = 0.25, power = 0.80) for a 2 (team type: human–AI vs. human–human, between-subjects) × 2 (misunderstanding type: information omission vs. ambiguous expression, within-subjects) mixed factorial design. The calculation indicated that a minimum total sample size of 128 participants was required to achieve adequate statistical power. The final sample size (*N* = 126) was therefore sufficient for hypothesis testing. The participants ranged in age from 18 to 25 years, with a balanced gender distribution (72 females, 57%; 54 males, 43%). All participants were randomly assigned to one of the four experimental conditions, with 32 individuals per condition, ensuring balance across groups. Informed consent was obtained prior to the experiment, and all recruitment and assignment procedures were conducted in accordance with ethical and scientific standards.

### 3.2 Experimental design and variable operationalization

This study employed a 2 (team type: human–AI vs. human–human, between-subjects) × 2 (misunderstanding type: information omission vs. ambiguous expression, within-subjects) mixed factorial design to systematically examine the effects of misunderstanding types and partner attributes on communication efficiency, team performance, and team trust. The core task involved cocreating content for a popular science WeChat public account focused on astronomy, a scenario chosen for its creativity, collaborative nature, and ecological validity. The task was pretested and validated for comprehension among the target population. Each dyad was required to complete three subtasks: (1) generate and name three column titles for astronomy-related science content, (2) design a theme and target audience for each column, and (3) collaboratively write a 500-word article for one selected column.

The experiment was conducted on an online platform, with all tasks delivered through a standardized digital interface to ensure consistency. In the human–AI condition, the system integrated DeepSeek—a generative AI with natural language understanding and reasoning capabilities—designed to simulate human-like collaboration. To enhance ecological validity, the AI agent provided dynamic textual feedback in response to participant input, rather than relying solely on pre-scripted responses. Nevertheless, certain aspects of the AI’s output remained partially scripted to maintain experimental control and consistency across sessions. The AI competence level was set to “moderate,” meaning that it could provide coherent outputs on the basis of logic and task demands but did not proactively initiate dialog or exhibit expert-level reasoning. “Moderate ability” was operationalized by benchmarking DeepSeek’s performance against standardized natural language tasks and expert ratings, ensuring consistent and reproducible outputs across sessions. All AI responses were generated from preset scripts and parameters to maintain consistent behavior across participants. The AI’s textual output was fully scripted and generated in a standardized format, following validated paradigms for low, medium, and high AI competence levels.

Misunderstanding type was treated as a within-subjects independent variable, with each participant encountering both information omission and ambiguous expression scenarios in a randomized and counterbalanced order to control for sequencing effects. Information omission refers to the deliberate or accidental exclusion of key information by the partner, leading to incomplete task comprehension (e.g., responding to only part of a proposed plan while ignoring other essential components). Ambiguous expression refers to vague or polysemous statements that obscure communicative intent, such as “This option still needs consideration” or “You should know what I mean,” creating uncertainty and interpretive confusion. Each misunderstanding type appeared an equal number of times (once per type per participant) and was embedded in scripted dialogs, uniformly presented across conditions to ensure comparable exposure and to elicit genuine cognitive dissonance and corrective efforts. In everyday communication, the frequency of information omission and ambiguous expression can vary depending on factors such as context, relationship, and topic. For instance, ambiguous expressions may be more common in informal conversations, while information omission might occur more frequently in task-oriented settings. In this study, both types were presented equally to allow for a direct and balanced comparison of participants’ responses to each misunderstanding type. This approach helps isolate the effects of misunderstanding type, though it may not fully reflect the nuanced distribution found in real-world interactions.

Peer type served as a moderator and referred to the identity of the participant’s teammate—either another human or an AI system. To avoid role confusion, each participant was exposed to only one peer-type condition throughout the experiment. Clear instructional prompts were used to prime perceptions of partner identity: in the human–human condition, participants were told that they were collaborating with another online participant (a trained confederate), whereas in the human–AI condition, participants were informed that their partner was an AI agent. A manipulation check at the end of the task asked participants to identify their collaborator, and only data from participants who answered this correctly were included in the final analysis.

Communication efficiency was assessed as a dependent variable using both objective and subjective indicators. Following the frameworks of Cooke et al. (2017) and Potter and Balthazard (2002), four metrics were used: (1) time to resolve misunderstanding, automatically recorded as the number of seconds between the introduction of a misunderstanding and the participant’s successful resolution; (2) number of communication turns, with more turns typically indicating greater difficulty in information exchange and consensus-building; (3) participant word count, calculated as the total number of words contributed across the dialog, reflecting both elaboration and potential redundancy; and (4) perceived communication efficiency, measured using a 5-point Likert scale capturing self-assessed clarity and fluency of interaction. The final submitted article was required to be 500 words in length. The word count for communication analysis only included the content generated during the dialog between participants and their partners, and did not include the collaboratively written article.

Team performance was assessed on the basis of the team’s ability to complete the collaborative task and the quality of the written output within the allotted time. The evaluation criteria were adapted from Kirkman et al. (2001) and Hoegl and Gemuenden (2001), with localization adjustments for the task context. Three expert raters independently scored each output on a scale of 0–10 for completeness, creativity, and practical relevance, and the interrater reliability was assessed using the intraclass correlation coefficient (ICC), which was 0.66 in this case. While an ICC of 0.66 indicates moderate reliability, it is consistent with prior studies involving subjective ratings of creative output (Stefanic and Randles, 2015). The average of the three ratings was used as the team’s performance score.

Team trust was assessed as a mediating variable, drawing on Mayer et al.’s (1995) model of organizational trust and the Trust-in-AI scale developed by Schaefer et al. (2016). In addition, ethical considerations were incorporated throughout the assessment process to ensure responsible research practices. Trust was measured using a five-item Likert instrument designed to evaluate the reliability, competence, and motivation of the partner (e.g., “I trust this AI teammate to complete the task,” and “I consider it a reliable partner”). To enhance the interpretability of the findings, trust was operationalized by focusing on participants’ perceptions of the AI teammate’s reliability, competence, and motivation, as captured by the specific items in the instrument. Both aggregate trust scores and subscale analyses were conducted to explore dimensional effects.

Two control variables were included to account for individual differences. First, participants completed the three-item Cognitive Reflection Test (CRT) developed by Frederick (2005) to assess rational reasoning and the ability to handle cognitively demanding problems (e.g., “A bat and a ball cost $1.10 in total. The bat costs $1 more than the ball does. How much does the ball cost?”). Second, participants completed the Meta AI Literacy Scale (MAILS) by Carolus et al. (2023), which evaluates AI familiarity and conceptual understanding (e.g., “I can explain the difference between artificial intelligence and traditional computer programs”).

### 3.3 Experimental procedure

The experiment was conducted in a controlled laboratory environment and lasted approximately 30 min. The main collaborative task, including all subtasks, took an average of 18 min to complete, while the posttask questionnaire required approximately 8 min. Each participant completed the survey once, immediately following the collaborative task. The procedure included four stages: check-in, task briefing, formal task execution, and posttask survey. Upon arrival, the participants completed registration and signed informed consent forms. The experimenter then delivered standardized instructions explaining the task background and platform operations while avoiding any disclosure of the partner’s true identity. During the main task, the participants completed the astronomy content creation assignment on the experimental platform with their assigned partner (human or AI). All interactions and timestamps were recorded by the system. After task completion, participants completed a posttask questionnaire assessing perceived trust in their partner, communication experience, satisfaction with team performance, and responses to manipulation check items.

## 4 Data analysis and results

### 4.1 Reliability, validity and manipulation checks

To ensure the robustness of the multivariate model, a comprehensive reliability and validity analysis was conducted. As shown in Table 1, all scales demonstrated high internal consistency, with Cronbach’s alpha coefficients ranging from 0.82 to 0.87—well above the recommended threshold of 0.70. The average variance extracted (AVE) for each construct exceeded 0.60, and the composite reliability (CR) values were all greater than 0.85, indicating satisfactory convergent validity and composite reliability. Effect sizes for group differences (Cohen’s *d*) were calculated for each scale to enhance interpretability of practical significance. As presented in Table 1, all effect sizes were small (*d* = 0.11–0.18), suggesting negligible baseline differences between team types. In addition, a series of one-way analyses of variance (ANOVAs) were performed to examine the mean differences in key variables across team-type conditions (human–human vs. human–AI). The results revealed nonsignificant differences in AI literacy (*F* = 1.12, *p* > 0.05), team trust (*F* = 1.05, *p* > 0.05), perceived communication quality (*F* = 1.19, *p* > 0.05), and team performance (*F* = 1.27, *p* > 0.05). Bonferroni correction was applied to control for Type I error inflation due to multiple comparisons; all results remained nonsignificant after adjustment (adjusted *p* > 0.05).

**Table 1:** Reliability, validity and descriptive statistics of scales.

Scale name	Cronbach’s *α*	AVE	CR	*F* Value	*p* Value	Cohen’s *d*
AI literacy	0.82	0.62	0.86	1.12	0.35	0.11
Team trust	0.84	0.65	0.88	1.05	0.38	0.12
Communication perception	0.86	0.68	0.89	1.19	0.31	0.15
Team performance	0.87	0.71	0.90	1.27	0.28	0.18

*F*-values and Cohen’s *d* were calculated based on team type groupings. Bonferroni correction applied.

**Figure 2:**
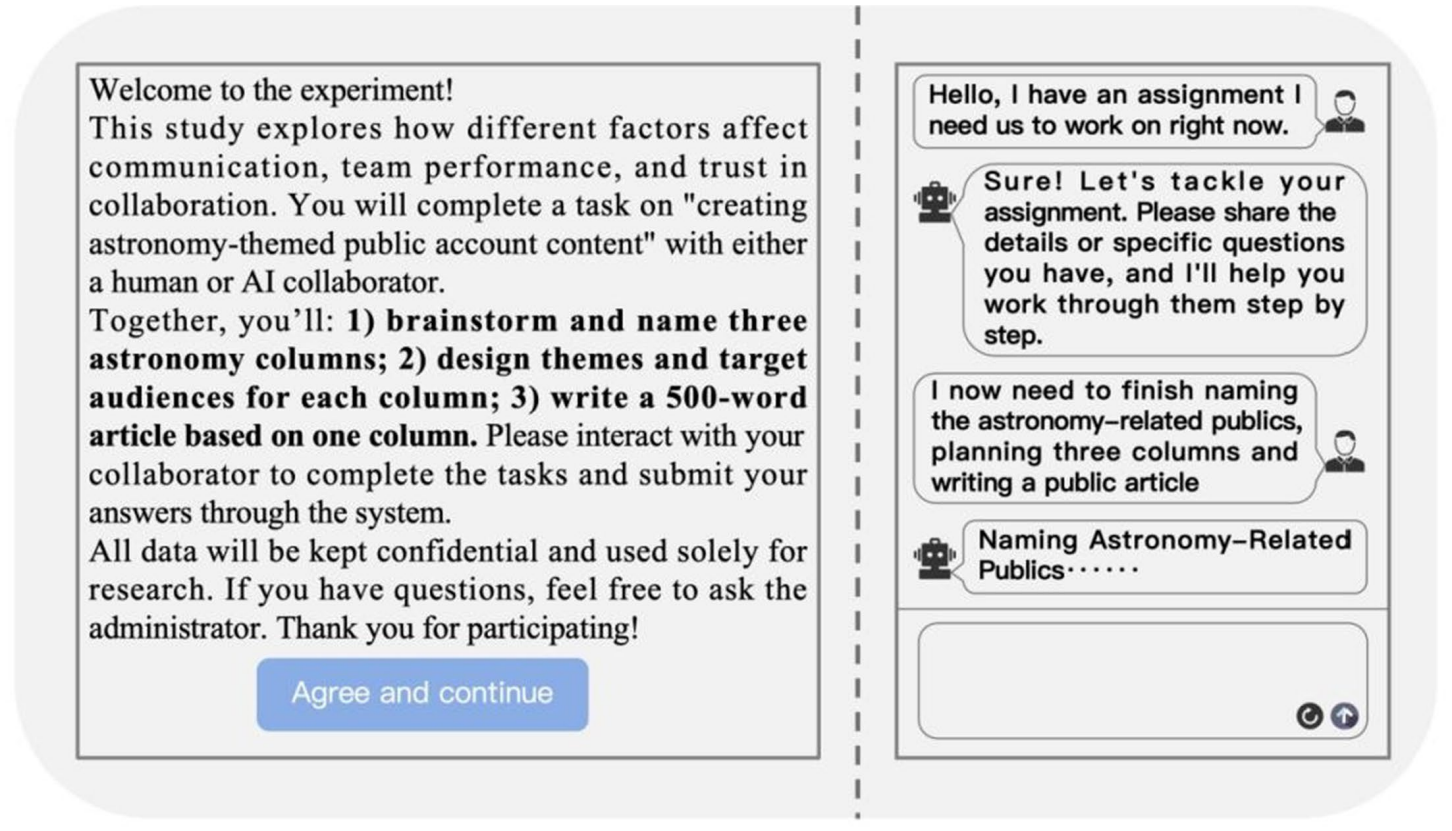
Experimental task interface and AI collaboration dialogue example.

Control variables, specifically Cognitive Reflection Test (CRT) and Multidimensional AI Literacy Scale (MAILS), were measured to account for participants’ cognitive style and AI-related knowledge. As shown in Table 2, although these variables were not included in the main regression models due to their nonsignificant associations with key outcomes, the regression coefficients for CRT and MAILS predicting communication perception (*β* = 0.049, *p* = 0.236; *β* = 0.056, *p* = 0.143), team performance (*β* = 0.037, *p* = 0.432; *β* = 0.062, *p* = 0.140), and team trust (*β* = 0.058, *p* = 0.138; *β* = 0.044, *p* = 0.237) were all small and nonsignificant. Sensitivity analyses further confirmed that including CRT and MAILS in the regression models did not substantially alter the main effects or model fit (Δ*R*^2^ < 0.01, all main predictors remained significant). This approach minimizes concerns about omitted variable bias and reinforces the robustness of the analytic strategy (Figure 2).

**Table 2:** Effects of control variables (CRT and MAILS) on main outcomes.

Dependent variable	Predictor	*β*	SE	*t* Value	*p* Value	95% CI
Communication perception	CRT	0.049	0.041	1.19	0.236	[−0.032, 0.130]
	MAILS	0.056	0.038	1.47	0.143	[−0.019, 0.131]
Team performance	CRT	0.037	0.047	0.79	0.432	[−0.056, 0.130]
	MAILS	0.062	0.042	1.48	0.14	[−0.021, 0.145]
Team trust	CRT	0.058	0.039	1.49	0.138	[−0.019, 0.135]
	MAILS	0.044	0.037	1.19	0.237	[−0.029, 0.117]

All models controlled for team type and misunderstanding type. None of the control variables reached statistical significance (*p* > 0.10), and inclusion did not substantially change the main effects reported in the primary analyses.

### 4.2 Correlation analysis

The study conducted correlation analyses for the main variables, as shown in Table 3. To examine the relationships among the main variables, correlation analyses were conducted, as presented in Table 2. The results demonstrate that both types of misunderstanding (information omission and ambiguous expression) show significant negative correlations with team trust (*r* = −0.181, *p* < 0.01), communication perception (*r* = −0.214, *p* < 0.01), and team performance (*r* = −0.162, *p* < 0.05). This indicates that higher levels of misunderstanding—regardless of type—are associated with lower team trust, less favorable communication perceptions, and decreased team performance. Further analysis reveals that team trust is positively correlated with communication perception (*r* = 0.633, *p* < 0.01) and team performance (*r* = 0.591, *p* < 0.01), suggesting that increased trust within teams facilitates better communication and enhances overall performance. In addition, communication perception shows a strong positive correlation with team performance (*r* = 0.727, *p* < 0.01), underscoring the importance of a positive communication climate in achieving team success. These significant correlations provide preliminary empirical support for the proposed hypotheses and justify subsequent regression analyses.

**Table 3:** Correlation analysis of main variables.

Variable	Mean	SD	1	2	3	4
1. Misunderstanding type	0.50	0.50	1			
2. Team trust	3.62	0.41	−0.181**	1		
3. Communication perception	3.71	0.42	−0.214**	0.633**	1	
4. Team performance	3.57	0.44	−0.162*	0.591**	0.727**	1

*p < 0.01, **p < 0.05.

### 4.3 Regression analysis

**Figure 3:**
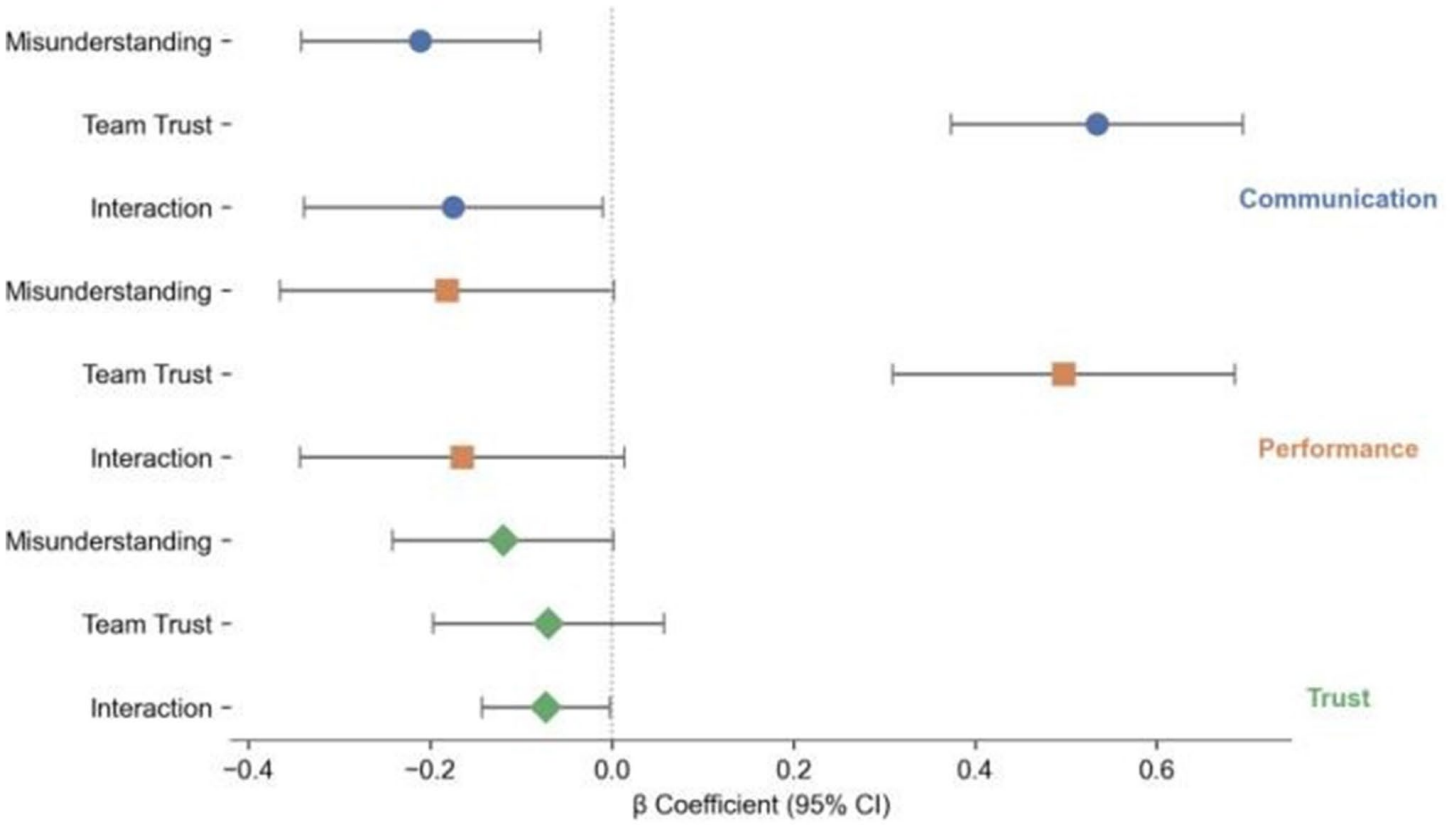
Regression analysis results for misunderstanding type, team trust, and team outcomes.

**Figure 4:**
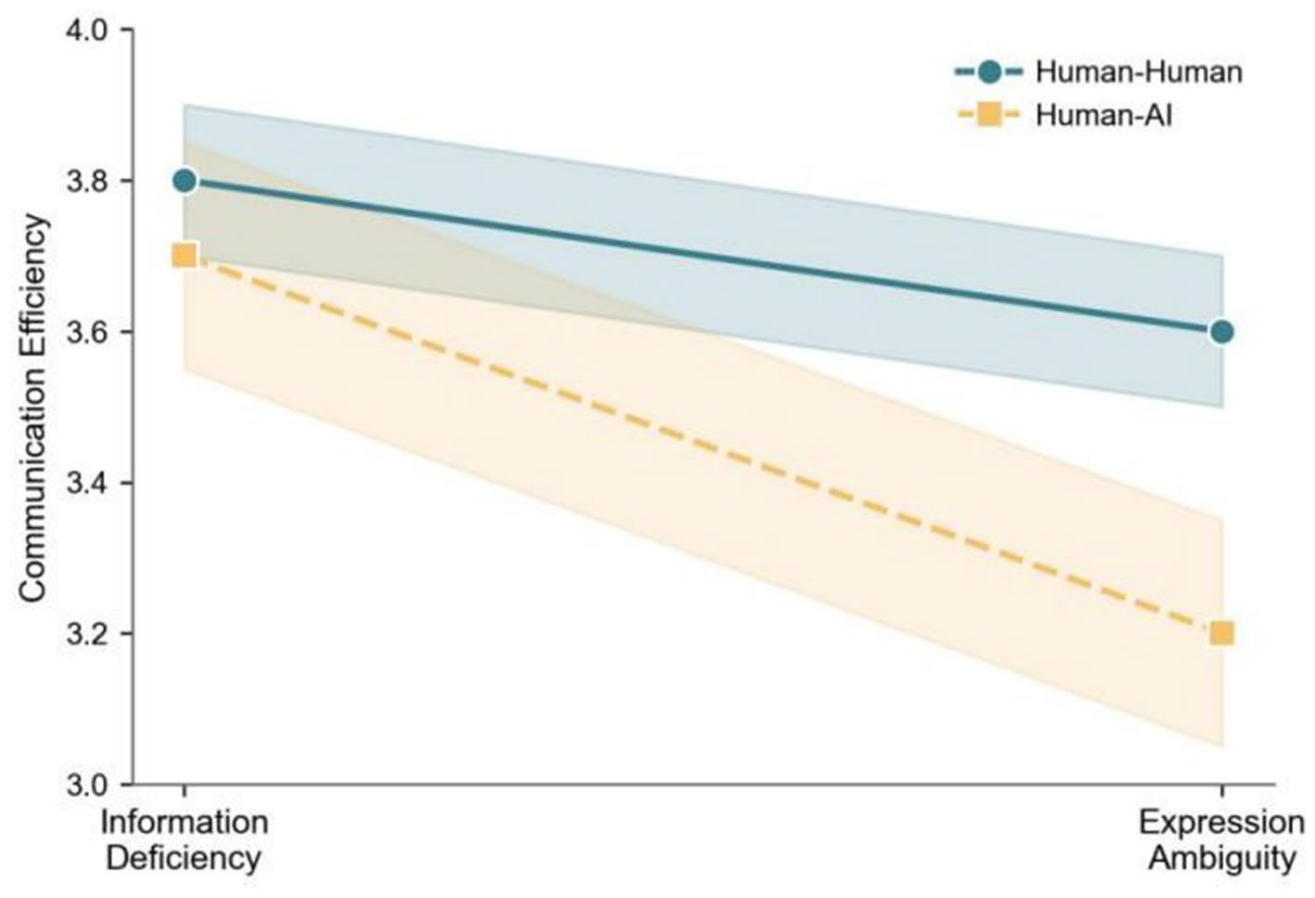
Interaction effects of misunderstanding type and team type on communication perception.

To systematically test our research hypotheses, we conducted multiple regression analyses. The results are summarized in Table 4 and Figures 3, 4, with effect sizes (Cohen’s *d*/*η*^2^) and post-hoc adjusted *p*-values provided for clarity.

**Table 4:** Regression analysis results (with effect sizes and post-hoc comparisons).

Dependent variable	Predictor	*β*	SE	*t* Value	*p* Value	Effect size (*d*/*η*^2^)	Post-hoc (*p* adj)
Communication perception	Misunderstanding type	−0.211	0.06	−2.63	0.01	*d* = 0.42	0.021
	Team trust	0.534	0.08	6.63	<0.001	*η*^2^ = 0.31	n.a.
	Team type	−0.142	0.09	−1.75	0.083	*d* = 0.15	n.s.
	Misunderstanding type × team type	−0.175	0.08	−2.13	0.036	*d* = 0.19	0.043
Team performance	Misunderstanding type	−0.182	0.09	−2.00	0.048	*d* = 0.37	0.049
	Team trust	0.497	0.09	5.44	<0.001	*η*^2^ = 0.28	n.a.
	Team type	−0.131	0.09	−1.44	0.156	*d* = 0.13	n.s.
	Misunderstanding type × team type	−0.165	0.09	−1.78	0.079	*d* = 0.16	0.081
Team trust	Misunderstanding type	−0.129	0.06	−2.00	0.047	*d* = 0.22	0.048
	Team type	−0.071	0.06	−1.17	0.244	*d* = 0.09	n.s.
	Misunderstanding type × team type	−0.073	0.03	−2.03	0.044	*d* = 0.10	0.045

n.a., not applicable; n.s., not significant.

Misunderstanding type significantly negatively predicted communication perception (*β* = −0.211, SE = 0.06, *t* = −2.63, *p* = 0.01, *d* = 0.42, *p* adj = 0.021) and team performance (*β* = −0.182, SE = 0.09, *t* = −2.00, *p* = 0.048, *d* = 0.37, *p* adj = 0.049). This indicates that ambiguous misunderstandings are more difficult to identify and resolve than information omissions, resulting in reduced communication efficiency and task completion. These findings support Hypotheses 1a and 1b. Team trust exerted a highly significant positive effect on both communication perception (*β* = 0.534, SE = 0.08, *t* = 6.63, *p* < 0.001, *η*^2^ = 0.31) and team performance (*β* = 0.497, SE = 0.09, *t* = 5.44, *p* < 0.001, *η*^2^ = 0.28), confirming that trust serves as a foundational mechanism for team collaboration. This supports Hypotheses 2a and 2b.

The moderation effects of team type (human–AI vs. human–human) were also examined. The interaction term (Misunderstanding Type × Team Type) had a significant negative effect on communication perception (*β* = −0.175, SE = 0.08, *t* = −2.13, *p* = 0.036, *d* = 0.19, *p* adj = 0.043). This suggests that ambiguous misunderstandings have a more pronounced negative impact on communication efficiency in human–AI teams. For team performance, the interaction effect was only marginally significant (*β* = −0.165, SE = 0.09, *t* = −1.78, *p* = 0.079, *d* = 0.16, *p* adj = 0.081), providing partial support for Hypothesis 3b, which claims stronger negative effects in human–AI teams. This result indicates that while the trend is in the expected direction, the statistical evidence is not robust enough to confirm a strong moderation effect for team performance. Additionally, the interaction term significantly predicted team trust (*β* = −0.073, SE = 0.03, *t* = −2.03, *p* = 0.044, *d* = 0.10, *p* adj = 0.045), suggesting that ambiguous misunderstandings are more likely to erode trust in human–AI teams. These results collectively support Hypotheses 3a, 3b (partially), and 3c.

### 4.4 Moderated mediation analysis

Prior to conducting moderated mediation analyses, we examined the assumptions of normality and linearity for the key variables. Normality was assessed using Shapiro–Wilk tests, while linearity was evaluated via inspection of residual plots for each regression model.

The Shapiro–Wilk test was conducted for all continuous variables involved in the mediation and moderation models, including communication perception, team performance, team trust, and misunderstanding type scores. The results are summarized in Table 5. All variables yielded *p*-values greater than 0.05, indicating no significant deviations from normality.

**Table 5:** Results of Shapiro–Wilk test for normality.

Variable	W Statistic	*p* Value
Communication perception	0.983	0.217
Team performance	0.979	0.138
Team trust	0.988	0.362
Misunderstanding type	0.985	0.254

All *p*-values > 0.05 indicate no significant departure from normality.

**Figure 5:**
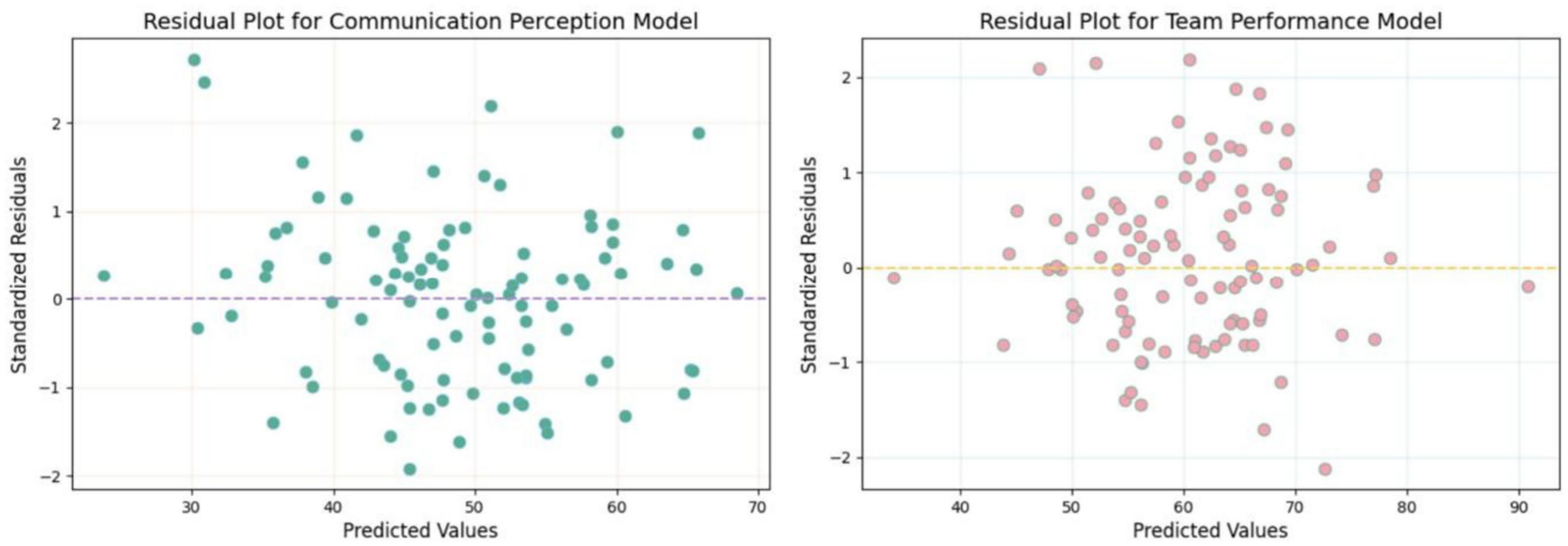
Residual plots for key regression models.

**Figure 6:**
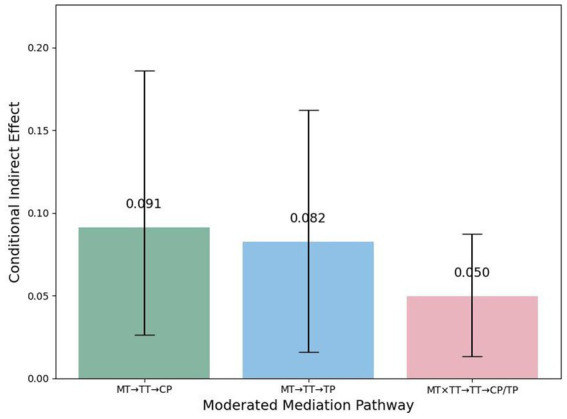
Moderated mediation pathways (Bootstrapped 95% CI).

Linearity was evaluated by plotting standardized residuals against predicted values for each regression equation. Visual inspection revealed no obvious curvilinear patterns or heteroscedasticity, supporting the assumption of linear relationships among the variables (Figures 5, 6).

Based on these results, the data satisfied the assumptions of normality and linearity, justifying the use of PROCESS Model 8 for subsequent moderated mediation analyses. As shown in Table 6, misunderstanding type had a significant indirect effect on both communication perception (moderated mediation effect = 0.0911, BootSE = 0.0434, BootLLCI = 0.0262, BootULCI = 0.1858) and team performance (moderated mediation effect = 0.0824, BootSE = 0.0461, BootLLCI = 0.0158, BootULCI = 0.1622) via team trust. Importantly, the direct effects of misunderstanding type on communication perception and team performance remained statistically significant after accounting for the indirect pathway through team trust (see Table 3), indicating that the moderated mediation effects were partially mediated rather than fully mediated. Furthermore, the interaction term of misunderstanding type and team type also exerted a significant moderated mediation effect via team trust on both communication perception and team performance (effect = 0.0498, BootSE = 0.0224, BootLLCI = 0.0134, BootULCI = 0.0873). These results provide robust support for Hypothesis 4, demonstrating that team type moderates the indirect pathway from misunderstanding type to team outcomes through team trust. Specifically, in human–AI teams, ambiguous misunderstandings more strongly undermine team trust, thereby amplifying their negative impact on communication perception and team performance.

**Table 6:** Moderated mediation analysis results.

Pathway	Moderator	Effect	BootSE	BootLLCI	BootULCI
Misunderstanding type → team trust → communication perception	Team type	0.0911	0.0434	0.0262	0.1858
Misunderstanding type → team trust → team performance	Team type	0.0824	0.0461	0.0158	0.1622
Misunderstanding type × team type → team trust → communication/performance	Team type	0.0498	0.0224	0.0134	0.0873

## 5 Discussion and implications

Drawing on evolutionary psychology and trust theory, this study systematically examined the effects of team type (human–AI vs. human–human) and misunderstanding type (information omission vs. ambiguous expression) on team communication efficiency, performance, and trust. Additionally, the moderating role of peer type and the mediating mechanism of trust were explored. The experimental results revealed that misunderstanding type significantly affected both communication efficiency and team performance, with information omission being easier for team members to identify and correct. Consequently, its negative impact on performance and communication was significantly smaller than that of ambiguous expression. These findings are consistent with prior research, which has demonstrated that information omission is typically more salient and thus more readily detected and addressed through clarification strategies (Brewer and Holmes, 2016; Laourou, 2022). In contrast, ambiguous expression often results in misinterpretation and prolonged communication breakdowns, as team members may not immediately recognize the misunderstanding (Sohrab et al., 2022; Boroomand and Smaldino, 2023). This aligns with the view that ambiguous misunderstandings are inherently harder to resolve due to their covert nature and the cognitive load required to identify and correct them (Paulus et al., 2024). Among all the experimental groups, ambiguous misunderstandings consistently produced stronger negative effects on communication and performance, especially in human–AI teams. Consistent with recent findings in evolutionary psychology (Heyes, 2020; Lew-Levy et al., 2023), the present results indicate that the effectiveness of social learning and meaning-making is contingent upon evolved cognitive mechanisms, which may be disrupted or attenuated within technologically mediated contexts. This suggests that ambiguity in AI-generated communication poses unique challenges, reinforcing the need for AI systems to incorporate mechanisms for ambiguity detection and resolution.

To further interpret these findings, we explicitly anchored our theoretical framework in evolutionary psychology—particularly the concept of evolutionary mismatch (Cosmides and Tooby, 1992; van Vugt et al., 2024). Human trust mechanisms evolved to assess reliability and intent based on rich social signals in face-to-face interactions. In human–AI teams, the absence of such cues—such as facial expressions, gestures, and vocal nuances—creates an evolutionary mismatch, making it challenging for team members to accurately calibrate trust in AI agents. As a result, ambiguous misunderstandings are more likely to erode trust, especially when social cues are missing. This perspective is supported by foundational work on cognitive adaptations for social exchange (Cosmides and Tooby, 1992), which highlights the role of evolved heuristics in reducing the risk of exploitation and facilitating cooperation (Capraro, 2024). Future models should explicitly consider the lack of evolved social cues in AI-mediated settings and examine how technology can simulate or supplement these cues to improve trust calibration.

Team trust emerged as a significant mediator in the relationship between misunderstanding type and both communication efficiency and performance. Information omission had a relatively limited impact on trust, as participants tended to engage in compensatory behaviors—such as using technical means or manual elaboration—thus maintaining stable trust dynamics. In contrast, ambiguous misunderstandings significantly reduce trust in the AI partner, which in turn impairs communication and task outcomes. This mediating effect aligns with recent calls in the literature to explore the underlying mechanisms of trust in collaborative work (Demir et al., 2021; Akiyoshi, 2022). Specifically, previous studies have shown that trust erosion following ambiguous misunderstandings is more pronounced when team members attribute errors to perceived limitations in AI’s language processing abilities (Zerilli et al., 2022; Zhang et al., 2023). From an evolutionary perspective, such attribution bias may reflect the lack of reputation management and social exchange cues in AI agents, which are critical for trust calibration in human interactions (van den Berg et al., 2024; Barclay, 2004; Lee et al., 2025). Marginally significant mediation effects further suggest that individual differences—such as prior AI experience or literacy—may modulate trust dynamics.

Peer type moderated both the direct impact of misunderstanding on team outcomes and the indirect effects mediated by trust. In human–AI teams, the detrimental effects of ambiguous misunderstandings on trust, communication, and performance are particularly pronounced. This is consistent with prior research highlighting attribution biases caused by AI’s “black-box” nature and communicative limitations (Zerilli et al., 2022; Zhang et al., 2023; Jackson, 2024). In contrast, human–human teams exhibit greater resilience when facing ambiguous misunderstandings, relying on a broader repertoire of strategies—including clarification, emotional cues, and context negotiation—to mitigate adverse effects. However, several moderation effects were only marginally significant. This result may reflect the influence of participants’ varying levels of prior experience with AI and differences in technical literacy. Individuals who are more familiar with AI systems or possess higher technical skills may find it easier to interpret and resolve misunderstandings, viewing such incidents as routine aspects of technology use rather than major obstacles. In contrast, those with less experience or lower technical literacy might perceive misunderstandings as more disruptive or challenging, which could impact their overall assessment of team performance and communication. Additionally, personal beliefs about technology, openness to innovation, and confidence in using AI tools could all shape how misunderstandings are processed and managed within a team context. Furthermore, evolutionary psychology suggests that individual variation in social learning and technological adaptation (Heyes, 2020; Lew-Levy et al., 2023) may shape how misunderstandings are processed and managed, highlighting the nuanced interplay between evolved cognitive mechanisms and modern collaborative contexts. These factors together may have contributed to the marginal significance observed in the moderation effects, highlighting the complex and nuanced ways that individual differences interact with human–AI collaboration.

When considering the repair of trust following AI errors, our findings and relevant literature suggest that trust can be partially restored if the AI subsequently provides a correct and timely response (Dietvorst et al., 2018). However, the extent of trust recovery depends on the perceived transparency of the AI’s corrective process and the frequency of previous errors. If the AI demonstrates consistent improvement and offers explanations for its corrections, participants are more likely to re-engage with the system and rebuild trust. According to evolutionary mismatch theory (Cosmides and Tooby, 1992; van den Berg et al., 2024), the lack of transparency and social feedback in AI agents undermines adaptive trust repair processes, which in ancestral environments relied on direct communication, emotional reassurance, and observable behavioral change. Conversely, repeated ambiguous misunderstandings without adequate repair mechanisms may lead to persistent trust deficits and reluctance to rely on AI partners.

Moderated mediation analysis revealed that in human–AI teams, ambiguous misunderstandings significantly amplified negative effects on communication and performance by eroding trust. In contrast, this indirect pathway was weaker in human–human teams. These findings deepen our understanding of the dynamic trust-mediation process in team collaboration and further underscore the contextual role of peer type (Edelmann et al., 2023). Human–human teams tend to possess stronger social bonds and shared communication norms, which facilitate the use of multimodal cues, real-time feedback, and clarification strategies to identify and correct misunderstandings. As a result, the deterioration of trust is less likely, and the downstream negative impacts on communication and performance are attenuated (Bahrain et al., 2023; Mohd Yusof and Zakaria, 2025). Moreover, members of human–human teams demonstrated greater tolerance for ambiguity and relied more on mutual assumptions of competence and positive intent (April et al., 2023). In contrast, human–AI teams are characterized by greater uncertainty and expectation gaps regarding AI capabilities, making ambiguous misunderstandings more likely to trigger trust crises and intensify negative outcomes (Edelmann et al., 2023). These patterns are consistent with evolutionary psychology’s predictions regarding the importance of social bonds and shared norms in facilitating trust and cooperation (Cosmides and Tooby, 1992).

From a theoretical perspective, this study extends existing models of team interaction by integrating the Input-Process-Output (IPO) framework and the Integrative Teamwork Competency (ITC) model (Körner et al., 2015; Roberts et al., 2022). The IPO framework emphasizes how shared context, communication processes, and adaptive coordination support effective collaboration, particularly in human–human teams where nonverbal cues and emotional intelligence play a critical role. In contrast, human–AI teams lack these adaptive mechanisms, resulting in fundamentally different trust dynamics and coordination breakdowns (Zhang et al., 2024). The ITC model further highlights the importance of mutual understanding and shared norms, which are less robust in human–AI collaborations due to the absence of genuine social bonds and contextual adaptation. By grounding our analysis in evolutionary psychology theory, we underscore the need for new models that address the unique mechanisms of trust formation and misunderstanding management in human–AI teams, especially considering the absence of evolved social cues and reputation management processes. Moreover, our findings resonate with evolutionary accounts of cultural learning and transmission, suggesting that AI-mediated teams may disrupt evolved processes for imitation, teaching, and peer learning (Boyer, 2000; Heyes, 2020; Lew-Levy et al., 2023).

In addition, factors such as individual cognitive style, AI familiarity, and communication preferences interact with misunderstanding type and overall communication effectiveness. For example, participants with higher AI literacy may be more adept at identifying and resolving ambiguous misunderstandings, while those with limited experience may be more susceptible to trust erosion and communication breakdowns (Dietvorst et al., 2018; April et al., 2023). These individual-level variables should be considered in future studies to develop a more comprehensive understanding of human–AI collaboration. Furthermore, our results indicate that organizational factors—including the establishment of standardized communication protocols, periodic training in human–AI collaboration, and the implementation of trust restoration mechanisms—play a pivotal role in mitigating the negative effects of misunderstandings. By linking these factors to misunderstanding management and overall team communication, organizations can foster more resilient and adaptive hybrid teams. Future research should investigate how technology can simulate or supplement missing social cues to improve trust calibration in AI-mediated settings.

Practically, this study offers actionable recommendations for both AI developers and organizational leaders. For AI designers, incorporating ambiguity detection and clarification features into the system architecture is crucial. For example, when multiple interpretations of user input are detected, the AI system should initiate clarification questions instead of providing potentially misleading responses. Interface design could also include “misunderstanding feedback” buttons, enabling users to flag ambiguous outputs and support ongoing system optimization. In unstructured tasks, AI agents should learn from historical communication data to adapt to individual linguistic preferences and minimize future misunderstandings. For managers, standardized communication protocols and clarification procedures should be established when AI is integrated into teams. Periodic training sessions focused on human–AI collaboration can enhance team members’ ability to detect and handle misunderstandings. Trust restoration mechanisms, such as retrospective review meetings following communication breakdowns, can be deployed to prevent escalation. Trust restoration mechanisms, such as retrospective review meetings following communication breakdowns, can be deployed to prevent escalation and facilitate the repair of team trust. Organizations should also invest in AI literacy training to help employees better understand how AI works, including its limitations and decision logic. These measures are essential for reducing ambiguity-induced communication breakdowns and trust crises, ultimately improving the efficiency and effectiveness of human–AI hybrid teams and supporting the broader goal of intelligent organizational transformation.

Despite its valuable contributions, this study presents several limitations that warrant consideration. First, the experimental task was centered on astronomy-related science communication, which, due to its specific nature, may constrain the generalizability of the findings to other collaborative domains. Future research should replicate and extend the proposed model in a variety of team contexts with increased complexity to enhance external validity. Second, the AI agent utilized in this study was scripted and preset regarding intelligence level and communication style. Although this approach facilitated controlled experimental conditions, it may not fully reflect the dynamic and multimodal interaction behaviors characteristic of real-world AI systems. Subsequent studies are encouraged to employ real-time AI agents or immersive technologies, such as virtual reality, to enable richer and more ecologically valid interaction scenarios, and to further investigate how the complexity of AI behavior influences team trust and communication processes. Third, the role of individual differences—including AI literacy, cognitive preferences, and prior experience—merits further exploration to understand how these factors interact with contextual variables in shaping trust dynamics and misunderstanding management. Future work should systematically examine these mechanisms and integrate AI design and trust-building strategies to advance a more comprehensive theory of human–AI collaboration. Additionally, issues related to AI transparency, fairness, and accountability should be addressed to mitigate potential algorithmic bias and trust crises. Fourth, trust was measured only after task completion, which may raise concerns about temporal precedence, as trust was modeled as a mediator of processes occurring during interaction. This post-task measurement approach was chosen to minimize disruption and to capture participants’ overall perceptions based on the entire collaborative experience. However, future research should consider employing multiple time-point measurements or process-tracing methods to better capture the dynamic development of trust throughout the interaction. Finally, the sample in this study had a relatively narrow age range (*M* = 22.5, SD = 2.7), which may limit the generalizability of the results. Future research should recruit participants from more diverse age groups to examine whether age moderates the observed effects.

## 6 Conclusion

Grounded in evolutionary psychology and trust theory, this study employed a 2 × 2 experimental design to systematically examine how team type (human–human vs. human–AI) and misunderstanding type (information omission vs. ambiguous expression) interact to shape communication efficiency, team performance, and team trust. The results indicate that ambiguous misunderstandings significantly undermine trust and collaboration in human–AI teams, primarily due to AI’s limited expressiveness and lack of transparency. Team trust emerged as a crucial mediating factor, with its effect most pronounced in human–AI contexts. These findings extend trust theory by highlighting the unique challenges posed by the absence of social signals and the moderating role of misunderstanding type in AI-mediated teams. In addition, the study demonstrates that different forms of misunderstanding have distinct impacts depending on team composition, suggesting that teams integrating AI require specific strategies to address ambiguity and foster clear communication. From an evolutionary perspective, trust functions as an adaptive mechanism to manage uncertainty and facilitate cooperation, and the lack of social cues in AI-driven interactions disrupts this mechanism, leading to trust miscalibration. Practically, organizations should enhance AI transparency, provide feedback mechanisms, and simulate social cues to foster trust and improve team outcomes. This research deepens our understanding of the psychological and relational dynamics underlying human–AI collaboration, and offers evidence-based recommendations for optimizing team performance in increasingly digital work environments.

## Data Availability

The raw data supporting the conclusions of this article will be made available by the authors without undue reservation.
